# Short-Term Effects of Ambient Air Pollution on Chronic Obstructive Pulmonary Disease Admissions in Jiuquan, China

**DOI:** 10.3390/toxics12050364

**Published:** 2024-05-15

**Authors:** Hairong Bao, Jiyuan Dong, Deshun Li, Lisha Zhu, Juan Shu

**Affiliations:** 1 Department of Gerontal Respiratory Medicine, The First Hospital of Lanzhou University, Lanzhou 730000, China; 2 School of Public Health, Lanzhou University, Lanzhou 730000, China

**Keywords:** Jiuquan, air pollution, COPD, DLNM, timeseries analysis

## Abstract

Recent findings indicate that air pollution contributes to the onset and advancement of chronic obstructive pulmonary disease (COPD). Nevertheless, there is insufficient research indicating that air pollution is linked to COPD in the region of inland northwest China. Daily hospital admission records for COPD, air pollutant levels, and meteorological factor information were collected in Jiuquan for this study between 1 January 2018 and 31 December 2019. We employed a distributed lag non-linear model (DLNM) integrated with the generalized additive model (GAM) to assess the association between air pollution and hospital admissions for COPD with single lag days from lag0 to lag7 and multiday moving average lag days from lag01 to lag07. For example, the pollutant concentration on the current day was lag0, and on the prior 7th day was lag7. The present and previous 7-day moving average pollutant concentration was lag07. Gender, age, and season-specific stratified analyses were also carried out. It is noteworthy that the delayed days exhibited a different pattern, and the magnitude of associations varied. For NO_2_ and CO, obvious associations with hospitalizations for COPD were found at lag1, lag01–lag07, and lag03–lag07, with the biggest associations at lag05 and lag06 [RR = 1.015 (95%CI: 1.008, 1.023) for NO_2_, RR = 2.049 (95%CI: 1.416, 2.966) for CO], while only SO_2_ at lag02 was appreciably linked to hospitalizations for COPD [1.167 (95%CI: 1.009, 1.348)]. In contrast, short-term encounters with PM_2.5_, PM_10_, and O_3_ were found to have no significant effects on COPD morbidity. The lag effects of NO_2_ and CO were stronger than those of PM_2.5_ and PM_10_. Males and those aged 65 years or older were more vulnerable to air pollution. When it came to the seasons, the impacts appeared to be more pronounced in the cold season. In conclusion, short-term encounters with NO_2_ and CO were significantly correlated with COPD hospitalization in males and the elderly (≥65).

## 1. Introduction

Chronic obstructive pulmonary disease (COPD) is a long-lasting respiratory condition marked by ongoing limitation of airflow, which is not completely reversible and develops progressively. The symptoms are mostly chronic cough, expectoration, and dyspnea, which may eventually lead to chronic respiratory failure [1]. COPD seriously affects patients’ labor ability and quality of life, brings certain mental and economic burdens to patients and their families, and causes certain social burdens, and it is a public health problem that attracts much attention today. According to World Health Statistics, in 1990, COPD was ranked as the twelfth leading cause of disability and the sixth leading cause of mortality; in 2020, it ranked as the fifth top cause of disability and the third top cause of death globally [2].

It has been reported that there were about 384 million people with COPD in the world in 2010, and the overall prevalence rate is about 11.7% [3]. The prevalence rate in developed countries is 3%, while the prevalence rate in some developing countries is far greater than 10% [4]. In recent years, the incidence and hospitalization rate of COPD in China have been on the rise. The survey shows that there are close to 100 million patients with COPD in China, with a prevalence rate of 13.7% among those aged 40 years and older [5], while this figure was only 8.2% ten years ago, suggesting that the prevention and control situation of COPD in China is grim [6].

The disease burden caused by respiratory diseases, mainly COPD, now ranks third among all kinds of disease burdens in China [7] and ranks first among all kinds of fatal diseases in rural areas [8]. Because of the high morbidity and mortality, the families of patients and society in general are spending more and more on COPD. COPD is a disease with complex pathogenesis, and it is influenced by individual susceptible factors such as genes, age, sex, lung growth and development, and environmental factors. Smoking (including active smoking and passive smoking) and biomass fuel burning are considered the most prominent causes of COPD [9]. Air pollution is the environmental factor with the widest exposure range, the longest duration, and the largest impact on people. Prior research has confirmed that exposure to outdoor air pollution is related to the incidence [9], but the research reports on the link between air pollution and COPD hospitalization are different at home and abroad. For example, an investigation conducted by Gao et al. revealed that significant effects on COPD hospitalization were observed for PM_2.5_, PM_10_, SO_2_, NO_2_, and CO but not for O_3_ [10]. In contrast, analyzing 255597 hospital admission visits in Qingdao, Yang et al. confirmed that only NO_2_ and SO_2_ exhibited distinct cumulative lag correlations with COPD admission [11]. Similarly, a study in Jinan, China, found that there was an absence of statistically significant variation in the health impacts induced by elevated levels of PM_2.5_, PM_10_, CO, and O_3_ [12]. In Istanbul, Turkey, Ko et al. found a direct correlation between air pollutants (PM_10_, PM_2.5_, and NO_2_) and an increase in hospital admissions for COPD [13]. As mentioned earlier, the majority of prior research on the relationship between outdoor air pollution and hospitalizations for COPD was conducted in eastern and central Chinese cities, including Beijing [10], Ningbo [14], and so on, where air contaminant concentrations and constituents were significantly different from those of cities in the inland northwest of China. In addition, most of the literature consists of studies on PM_10_, SO_2_, and NO_2_, while PM_2.5_ and CO, which may have a greater health impact, have received less attention. Therefore, more and more in-depth research is needed to discover the role of environmental factors, especially air pollution, in the formation and pathogenesis of COPD and to establish a foundation for developing prevention and control policies and measures that effectively combat COPD in China.

In China, greater emphasis was placed on the prevention and management of air pollution in heavily polluted cities, such as Beijing, compared to cities with lower pollution levels, even though some publications suggested that cities that had lower air pollution levels indicated higher risks of COPD [14,15].

During the period of 2018–2019, the daily average concentration of PM_2.5_, PM_10_, SO_2_, NO_2_, O_3_8h (maximum values of 8-h moving average for ozone), and CO in Jiuquan was 25.74 μg/m^3^, 78.41 μg/m^3^, 10.91 μg/m^3^, 22.55 μg/m^3^, 100.11 μg/m^3^, and 0.57 mg/m^3^, respectively. These values for PM_2.5_, PM_10_, NO_2_, and O_3_8h exceed the newly revised Global Air Quality Guidelines 2021 of the World Health Organization (annual average, PM_2.5_: 5 μg/m^3^; PM_10_: 15 μg/m^3^; SO_2_: 40 μg/m^3^; NO_2_: 10 μg/m^3^; O_3_8h: 100 μg/m^3^; CO: 4 mg/m^3^), except for SO_2_ and CO. Hence, our attention was drawn to cities such as Jiuquan that exhibit comparatively low levels of pollution.

To provide local updated evidence and fill the gaps in knowledge, we adopted the DLNM to assess the relationship of six air contaminants on hospitalizations for COPD in Jiuquan, a city located in the interior northwest of China, and utilized stratified analyses to see how the effects varied according to gender, age, and season.

## 2. Data and Methods

### 2.1. Study Area

**Figure 1:**
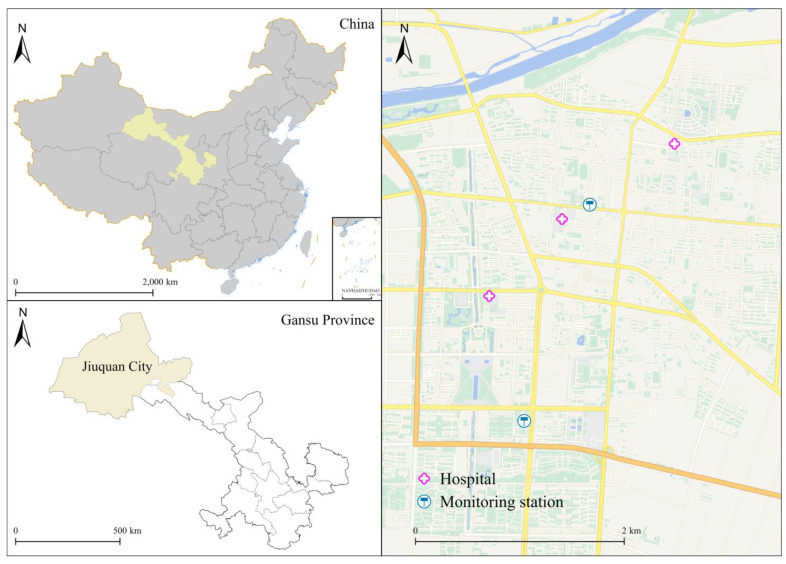
Geographical location of Jiuquan, China.

Jiuquan (38°05′~42°43′ N, 92°23′~100°21′ E) lies in the western part of Gansu Province, China (Figure 1), with approximately 450,000 permanent residents in 2019. The research region features a cold-temperate desert environment characterized by dry and chilly winters and scorching summers. The average yearly temperature and relative humidity are 8.18 °C and 46.49%. With the improvement in the quality of life in Jiuquan, the number of private cars has risen sharply and urban transportation is facing great pressure, while vehicle exhaust emissions have caused serious air pollution problems.

### 2.2. Data Collection

In our study, we collected the daily COPD hospital admission data for the years 2018 to 2019 from all local public hospitals (Jiuquan First People’s Hospital, Jiuquan Second People’s Hospital, and Jiuquan Hospital of Traditional Chinese Medicine) in Jiuquan. The COPD inpatient records included gender, age, admission date, and main diagnosis (code: J40-J44) based on the International Classification of Diseases, 10th revision (ICD-10) codes.

The hourly concentrations of ambient air pollutants, which include fine particulate matter (PM_2.5_), inhalable particulates (PM_10_), sulfur dioxide (SO_2_), nitrogen dioxide (NO_2_), ozone (O_3_), and carbon monoxide (CO), from monitoring stations were obtained from the Jiuquan Ecological Environment Bureau throughout the study period. As Jiuquan is located in a semi-arid desert oasis, the urban area is small and the population is concentrated, according to the requirements for monitoring stations in China’s ambient air quality standards (GB 3095-2012), two local monitoring points in Jiuquan can meet the actual needs of air quality monitoring and better reflect the air pollution situation in the whole city (see Figure 1). Based on the Chinese government’s technical requirements, two local monitoring points are located distant from traffic crossings, significant industrial pollutants, and any other polluting sources. The measures for quality assurance/quality control (QA/QC) were properly implemented according to two standards (HJ 653-2013 and HJ 654-2013) issued by the Ecology and Environment of the People’s Republic of China. In addition, three hospitals and two monitoring stations are located within a 5–15 km radius of each other (see Figure 1), which can adequately represent the population’s exposure level. Then the 24 h average concentrations of PM_2.5_, PM_10_, SO_2_, NO_2_, and CO were calculated and represented as daily average concentrations, and the O_3_ concentration was an 8 h maximum value concentration (O_3_8h) from all verified monitoring locations. Similar to the methods used in a number of earlier studies [10,11,12,14,15], we calculated the average air pollution concentrations from the 2 monitoring sites to represent the overall exposure for all people. Daily meteorological information, which comprised temperature and relative humidity, was supplied by the China Meteorological Data Service Center (http://data.cma.cn/, accessed on 12 March 2020). This research has complete data for all six air contaminants and climatic indicators.

### 2.3. Statistical Analysis

Due to the low frequency of daily hospital admissions for COPD in comparison to the local population, they follow a Poisson distribution. Furthermore, because the distribution of hospital COPD admissions was “over-dispersion”, a quasi-Poisson distribution was finally adopted [14]. The impact of air pollutants on COPD has often exhibited evident hysteresis, which signifies a postponement of the deleterious consequences of air pollutants. The maximum effect is typically observed several days after the onset of cumulative exposure. Therefore, we used the quasi-Poisson generalized additive model (GAM) with a distributed lagged nonlinear model (DLNM) to assess the association between six air pollutants and hospital admissions for COPD. Concerning the influence of confounding variables such as weather conditions, time variables were controlled by the natural cubic spline (*ns*) function. Additionally, the impact of weekends and public holidays was adjusted for by y incorporating the terms “*Holiday*” and “*Dow*” into the model, respectively. Two variables with a correlation coefficient below 0.7 were excluded from the same model in order to prevent multicollinearity, as determined by Spearman’s analysis. The model that incorporates covariates was built as follows:

Log(*u_t_*) = *α* + *βX*_*t*,l_ + *ns*(*Tem_t_*, *df*) + *ns*(*rh_t_*, *df*) + *ns*(*Time_t_*, *df*) + *factor*(*Dow*) + *factor*(*Holiday*) (1)

where *u_t_* refers to the observed daily number of hospital COPD admissions on day *t. t* represents the day of observation; *α* is the intercept, *β* is the vector of coefficient; *l* is the lag day; and *X* represents the dlnm cross basis matrix of six air pollutants, and we fitted the exposure–response and lag–response correlations between air pollution and COPD admissions by using a linear and natural cubic spline (*ns*) function. *df* is the degrees of freedom of natural cubic spline function. *Tem_t_* is the daily average temperature on day *t*; *rh_t_* is the daily average relative humidity on day *t*; and *Time_t_* is the calendar time, which is used to control the long-term trend and seasonality. *Dow* is the indicator variable to control variations among a day of the week; *Holiday* is the indicator variable to control Chinese public holidays. Degrees of freedom (*df*) for each variable were determined according to the Akaike information criterion for quasi-likelihood models (QAIC). We evaluated the optimal *df* for time with 7, for temperature and relative humidity with 3 to control the long-term and seasonal trends and the effect of temperature and relative humidity.

Previous studies showed that the delayed effects of air pollution on COPD morbidity could last for several days, and the biggest air pollution lag effects occur within 7 days [10,11,12,14]. Therefore, we decided to use a maximum lag of 7 days in the DLNM for each pollutant, as in the three documents mentioned above [10,11,12,14]. Research has indicated that the cumulative effect in the single-pollutant model may be underestimated [14]. So, we not only applied single lag days (lag0–lag7 day) but also incorporated the multiday moving average lag of lag01–lag07 to investigate the lagged associations. 

Consistent with a typical approach that was utilized in two earlier studies [11,14], we selected the lag day with each pollutant that produced the highest RR in the single-pollutant model to conduct subgroup analyses stratified by gender (male vs. female), different age (<65 years vs. ≥65 years), and season (hot season: April–September, cold season: October–March) on COPD admissions. We also fitted the exposure–response curve and conducted sensitivity analysis based on the lag day with each pollutant that produced the highest RR in the single-pollutant model.

To evaluate model robustness, first, we built two pollutant models to assess the confounding effects. Second, additionally, the df was modified for the temporal trends (6–10 df/year).

The relative risk (RR) and 95% confidence interval (95% CI) in COPD admissions were calculated for each 10 μg/m^3^ increase in the concentrations of PM_2.5_, PM_10_, SO_2_, NO_2_, and O_3_8h (except for each 1 mg/m^3^ increase in the concentration of CO). For the purpose of comparison with other studies, we also characterized the hazards of air pollutants in the discussion using percentage change (PC): PC = (Relative Risk − 1) × 100%. All analyses were performed by using the “mgcv” and “dlnm” software packages in R 4.0.2.

## 3. Results

Table 1 displays the statistical characteristics of daily hospital admissions for COPD, weather conditions, and air pollutants in Jiuquan from 2018 to 2019. The study incorporated a total count of 8367 hospital admissions for COPD, with a daily average of 8 cases. Among all the inpatients, 53.94% were females, and 67.22% were aged 65 years and above. The average daily concentrations of PM_2.5_, PM_10_, SO_2_, NO_2_, O_3_8h, and CO were 25.74 μg/m^3^, 78.41 μg/m^3^, 10.91 μg/m^3^, 22.55 μg/m^3^, 100.11 μg/m^3^, and 0.57 mg/m^3^, respectively. The daily mean temperature was 8.18 °C, and the relative humidity was 46.69%.

Table 2 displays the Spearman correlation between ambient air pollutants and weather conditions. Except for O_3_8h and relative humidity, the rank correlation coefficients between all two variables in this study are of statistical significance (*p* < 0.05). The daily concentrations of PM_2.5_, PM_10_, SO_2_, NO_2,_ and CO were significantly positively correlated, with PM_2.5_ and PM_10_ being the most pronounced (*r* = 0.85). Conversely, O_3_ had negative correlations with other air contaminants (−0.39 ≤ *r* ≤ −0.17). The temperature was negatively correlated with all the air pollutants (−0.56 ≤ *r* ≤ −0.25) except O_3_8h (*r* = 0.80). Moreover, the relative humidity showed a positive correlation with PM_2.5_, NO_2,_ and CO (0.15 ≤ *r* ≤ 0.29) but a substantial negative correlation with O_3_8h (*r* = −0.33).

**Figure 2:**
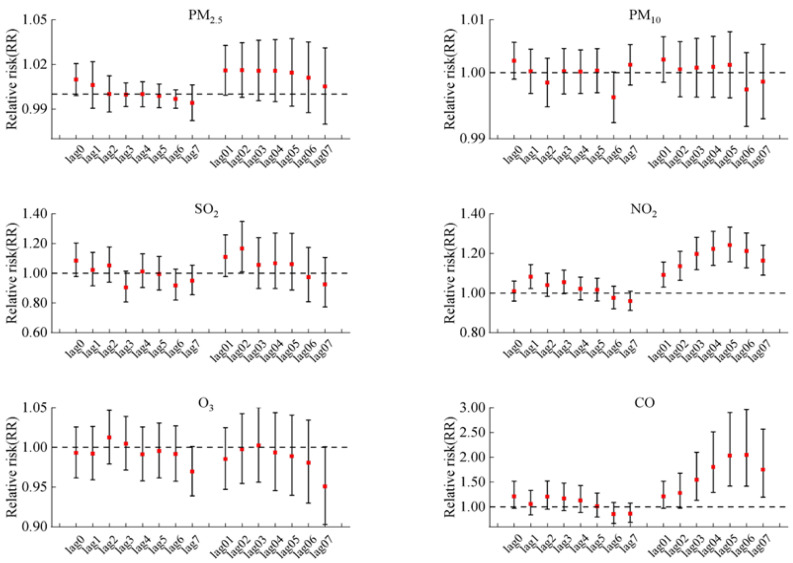
The RR with 95% CIs of COPD hospital admissions for each 10 μg/m^3^ increase in air pollutants (1 mg/m^3^ in CO) in single-pollutant models at lag day 0–7 and at cumulative lag day 01–07.

Figure 2 illustrates the delayed impact for every 10 μg/m^3^ increase in PM_2.5_, PM_10_, SO_2_, NO_2_, and O_3_8h concentration and for 1 mg/m^3^ increase in CO concentration on COPD hospitalizations in the single-pollutant model. And the lag patterns for the six air pollutants varied generally. We found significant associations between hospitalizations for COPD and NO_2_ in lags of 1 and all cumulative lag days (lag01–lag07). A significant correlation was also observed between COPD hospitalizations and CO on lag03 to lag07 days. The biggest adverse effect in single-pollutant models was at lag05 with a risk rate (RR) = 1.015, 95% confidence interval (95% CI: 1.008–1.023) per 10 μg/m^3^ increase in NO_2_, and RR = 2.049 (95% CI: 1.416–2.966) per 1 mg/m^3^ increase in CO, respectively. In addition, SO_2_ just exhibited a harmful impact at lag02 (per 10 μg/m^3^ increase, RR = 1.167, 95% CI: 1.009–1.348). In contrast, short-term exposure to PM_2.5_, PM_10,_ and O_3_ had no significant impacts on COPD morbidity.

**Figure 3:**
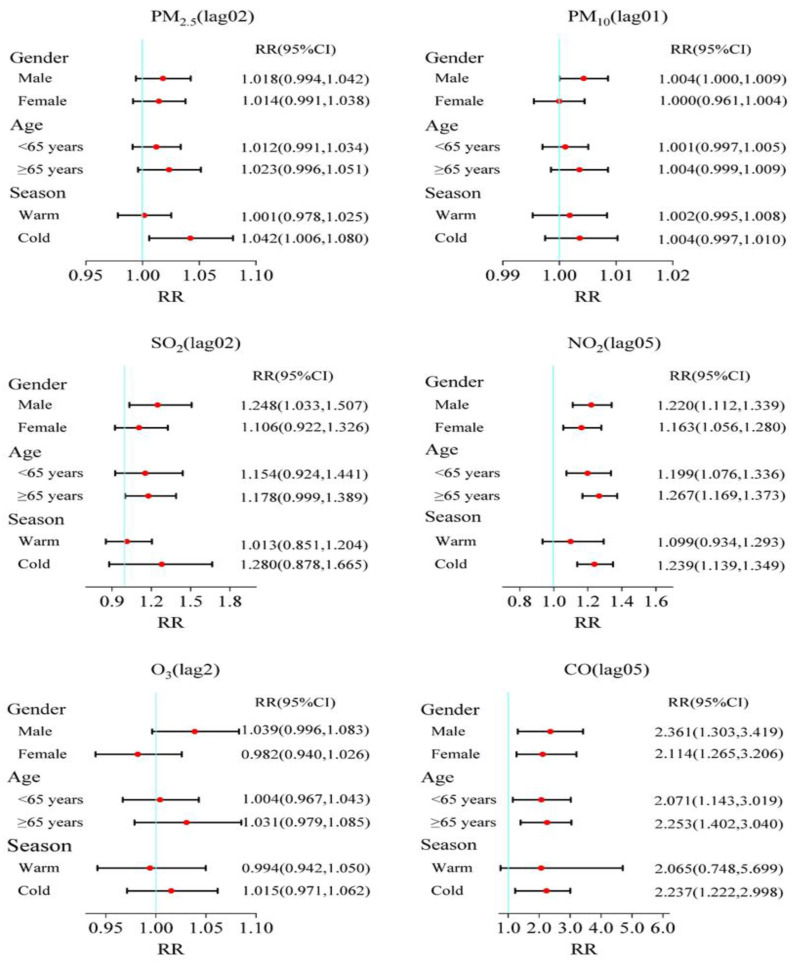
RR and 95% CI of COPD hospitalizations with an increase of 10 μg/m^3^ in air pollutants (1 mg/m^3^ in CO) stratified by gender, age, and season at its maximum lag days in Jiuquan, China.

Figure 3 illustrates the subgroup analysis results of different genders, ages, and seasons. Overall, the associations between air pollutants and COPD admissions appeared to be more evident for males, the elderly, and in cold seasons. For different population groups, positive correlation results were observed for male patients with PM_10_, SO_2_, NO_2,_ and CO and had significant associations in both male and female groups, and the effects estimates were also higher with the male patients than the females. Significant correlations were discovered for people aged ≥65 years in NO_2_ (RR = 1.267, 95%CI: 1.169, 1.373) and CO (RR = 2.253, 95%CI: 1.402, 3.040); no significant associations were observed for all age groups for the four other pollutants. The situation, on the other hand, was distinct in the cold season. Only PM_2.5_, NO_2_, and CO were found to be significantly associated with an increase in COPD hospitalizations during the cold season.

**Figure 4:**
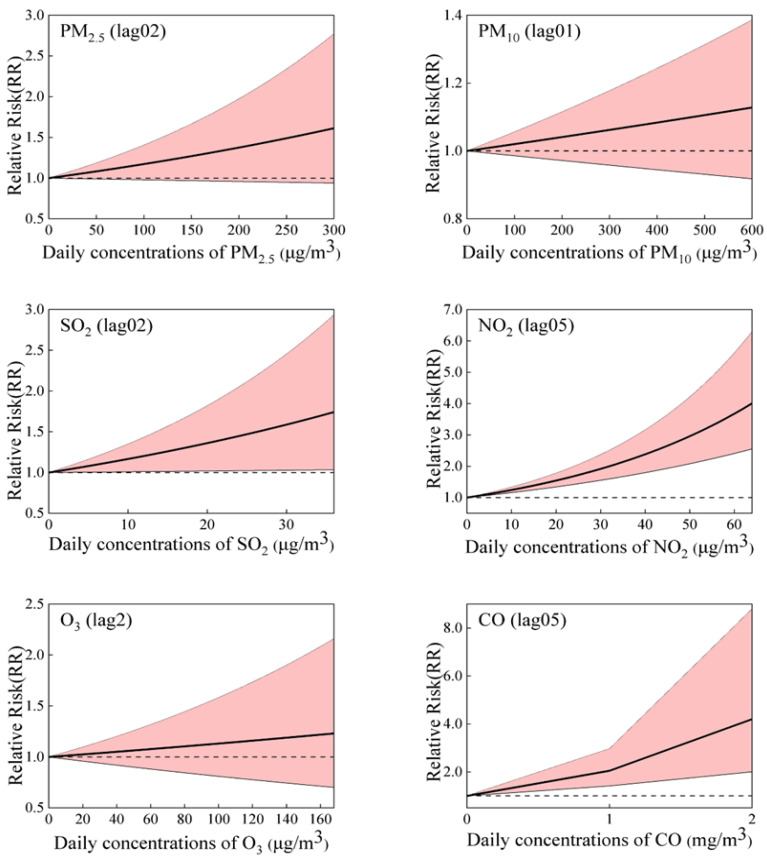
The exposure–response (E-R) curves for the correlation between six air pollutions and COPD hospital admissions.

Figure 4 shows the exposure–response curve correlating six air pollutants with the relative risk (RR) for COPD morbidity. As illustrated in the figure, we found a linear correlation between all the pollutants and COPD hospitalization, without any discernible thresholds indicating a significant influence, even at extremely low concentrations. The relationships of air pollutants with COPD hospitalization were positive and statistically significant for SO_2_, NO_2_, and CO, even at lower than relevant limit values of air quality standards (150 μg/m^3^, 80 μg/m^3^, and 4 mg/m^3^ for daily mean SO_2_, NO_2_, and CO, respectively). We detected no significant association between PM_2.5_, PM_10_, and O_3_8h levels and the risk of hospitalization for COPD.

Figure S1 depicts the outcomes of single- and double-pollutant models on the best lags. The estimated effect of each pollutant remained unchanged after the adjustment for additional air pollution. Table S1 shows that the association between pollutants and COPD hospitalization was robust when altering 6–10 degrees of freedom for time.

## 4. Discussion

Our study’s results demonstrated a strong correlation between COPD admissions and exposure to the pollutants SO_2_, NO_2_, and CO, except that PM_2.5_, PM_10,_ and O_3,_ and these three gaseous pollutants had a bigger effect on COPD admissions than particulate air pollution. It was also found that the effect of NO_2_ and CO on COPD with a cumulative lag was stronger than the effect with a single lag. Additionally, the associations were stronger in females, patients aged 65 and above, and in cold seasons. Moreover, the exposure–response relationship curves of air pollutants were almost linear.

Unfortunately, our findings for particulate matter do not align with several previous reports in the literature that have been conducted in some other regional countries or cities [13,14,15,16,17,18,19]. We observed that short-term exposure to PM_2.5_ or PM_10_ did not correlate significantly with COPD morbidity, which was consistent with findings from Berlin, Germany, and Jinan, China, the authors of the above two articles identified that PM_2.5_ and PM_10_ were also not found to significantly influence COPD hospitalizations in their study [12,20], while the results of some prior studies in Zhangjiakou, China; Zigong, China; Beijing, China; Ganzhou, China; Ningbo, Guangzhou, China; Gyeonggi-do, South Korea; and Istanbul, Turkey, found a statistically significant positive relationship between PM_2.5_, PM_10_, and COPD morbidity [13,14,15,16,17,18,19,20,21]. A recent systematic review, comprising 19 timeseries studies from around the globe, also estimated that the same PM_2.5_ increase would result in a 1.60 percent increase in COPD admissions [22]. The reasons for these inconsistent results are mainly due to the fact that Jiuquan has taken a series of local measures to control and manage particulate pollution. For example, Jiuquan implemented a comprehensive modification of its emergency air pollution reduction inventory in mid-2017, resulting in more efficient methods to decrease particle matter. The main measures consisted of the following: implementation of industrial restructuring requirements, systematic execution of the conversion of energy-intensive sectors into ultra-low emission systems; continuing to control volatile organic compound (VOC) emissions; improving dust control; speeding up the treatment of diesel trucks; and rigorously regulating total coal consumption.

However, in comparison to other studies, for a 10 µg/m^3^ increase in SO_2_, our estimates of 16.7% for COPD morbidity were higher than the estimates noted by different countries, regions, or cities around the world. For example, Yang et al. reported that a 10 μg/m^3^ increase in SO_2_ was associated with a 4.6% increase in COPD admissions at lag07 [16]. Ding et al. reported that during 15 days after exposure, a 10 μg/m^3^ increase in SO_2_ was correlated with a 0.37% increase in COPD admissions [17]. Some other timeseries studies have also discovered a stronger link between short-term SO_2_ exposure and COPD hospitalization, and the percentage change (PC) was 2.10% for Beijing [10], 1.60% for Qingdao [11], 5.20% for Ningbo [14], and 2.39% for Jinan [12]. The observed heterogeneity can be ascribed to variations in climatic conditions, sample populations, and study designs. For example, in autumn and winter in Jiuquan, due to the obvious cooling of the ground by radiation at night, an “inversion layer” is likely to appear in the middle and low altitudes of the atmosphere. This leads to a weakening of the ability of air to exchange and circulate horizontally and vertically. Pollutants emitted from the air are confined to the shallow atmosphere and gradually accumulate, leading to air pollution.

Our findings suggested that O_3_ exposure had no significant effect on COPD admissions. Two other timeseries analyses conducted in Ningbo and Jinan, respectively, found no statistically notable correlation between O_3_ exposure and COPD admissions [12,14]. However, the findings of a few other studies were not in agreement with the results of this investigation, and there was a positive association between O_3_ and COPD admissions [15,16,17]. One reason for the differences in the study data could be that ozone (O_3_) production mechanisms are complex. It should be noted that ozone (O_3_) exhibits high chemical reactivity and readily undergoes reactions with other atmospheric contaminants, resulting in the formation of novel molecules and making them unstable in the environment. As a result, we ought to exercise caution while elaborating on the consequences of being exposed to oxygen. There is still a need for more research into the mechanism of O_3_ in COPD.

In the current study, NO_2_ and CO were slightly greater than PM_2.5_ or PM_10_ to be connected to the risk of COPD, and our results showed that a 10 μg/m^3^ increase of NO_2_ and 1 mg/m^3^ CO was associated with an increase of 15.0% and 104.9% in COPD hospital admissions, respectively. Similar to our research results, several previous studies in China investigated the relationship between NO_2_ and CO and COPD admissions and reported a strong association between NO_2_ and CO and COPD admissions [11,16,17,18]. For example, a study conducted in the inland city of Zhangjiakou, China, reported that a 10 µg/m^3^ increase in NO_2_ and 1 mg/m^3^ CO was substantially associated with 10.2% and 0.20% increases in daily COPD morbidity [16]. An additional timeseries analysis conducted in Zigong, China, revealed a correlation between short-term exposure to NO_2_ and CO and a greater risk of COPD morbidity; a 10 µg/m^3^ increase in NO_2_ and 1 mg/m^3^ increase in CO corresponded to an increased number of COPD morbidity at 0.2% and 0.26% (Ding et al. 2018). Gao et al. reported that an increase in NO_2_ (10 µg/m^3^) and CO (1 mg/m^3^) was connected with an increase of 3.0% and 6.0% in COPD morbidity in Beijing, China [18]. Yang et al. reported that an increase of 10 μg/m^3^ in NO_2_ and 1 mg/m^3^ CO corresponded with an increase in COPD morbidity of 1.36% and 0.05%, respectively, in Qingdao, China [11]. The effect estimates in this study were significantly greater than the values of the four papers mentioned above. The large effect size in this study might be explained by several reasons, including (a) Vehicle emissions: With the improvement of people’s quality of life, the number of private cars has risen sharply, urban traffic is under great pressure, and vehicle emissions have caused serious air pollution problems. (b) Unfavorable meteorological conditions: In autumn and winter, as a result of significant radiative cooling of the ground at night, an “inversion layer” tends to appear in the atmospheric mid and lower regions, resulting in weaker horizontal and vertical exchange and circulation of air. Pollutants emitted from the air are confined to the near-surface atmosphere and gradually accumulate, leading to air pollution.

To investigate the possibility of modifiable factors that might influence the link between air pollution and COPD, we carried out some subgroup analyses. In terms of sex, we found that males had a higher vulnerability to air pollution compared to females. A comparable investigation conducted in Beijing, China, also concluded that increased air pollution significantly increases the risk of COPD admissions for males [18]. In comparison, the research conducted by Yang et al. [11] and Cheng et al. [12] demonstrated that air pollution had a statistically significant effect on COPD in females but not in males. From this, we can see that existing findings on gender disparities in the epidemiology of air pollution yielded inconsistent. The range of possible factors that influence gender differences is quite large, which may lead to inconsistency in the findings. The disparity could be due to various factors, including, but not limited to, sex-related biological variations, hormonal state, occupational exposures, smoking behavior, home exposures, and even differing responses to things that cause stress. In regard to age, individuals aged 65 and above were found to be more vulnerable to air pollution and, consequently, were more likely to suffer from COPD, as indicated by certain studies conducted in China and Korea [12,14,18]. The heightened vulnerability of elderly individuals to the impacts of air pollutants may be related to the fact that aging and comorbidity could weaken immune defenses and respiratory function, resulting in respiratory infections. According to our investigation, the risk estimate was higher in the cold season compared to the warm season. The result is in line with relevant research in Qingdao [11]. However, our findings were different from those of others, suggesting more pronounced harmful effects during the warm season [14,18]. The reasons for this are as follows: Jiquan is located in the interior of northwest China, with low average temperatures, where fossil fuels like coal are employed for heating buildings during a 5-month period annually. In autumn and winter, as a result of significant radiative cooling of the ground at night, an “inversion layer” tends to appear in the atmospheric mid and lower regions, resulting in weaker horizontal and vertical exchange and circulation of air. Pollutants emitted from the air are confined to the near-surface atmosphere and gradually accumulate, leading to air pollution. Cold air can also provoke pain in persons with COPD by stimulating the airway.

Concerning the delayed impact of air pollution on COPD morbidity, our findings demonstrated a notable cumulative delayed impact of NO_2_ and CO on COPD, and the effects of moving average lags were higher than single lags in various time periods, which was essentially consistent with another study focusing on Beijing, China [10]. Variations in cumulative lag days and effect sizes may be due to the physicochemical qualities of the pollutants and the biological mechanisms involved. For example, studies have shown that when the body is exposed to NO_2_ and CO, it stimulates airway hyperresponsiveness and induces an inflammatory response in the body, resulting in macrophage and epithelial cell dysfunction [22,23,24,25]. These effects, in turn, exacerbate the airway inflammatory state in patients with chronic obstructive pulmonary disease [26]. The development of these processes required a period of time, after which cumulative effects were produced.

Our study has limitations. First, individual exposure in the current study was replaced by the average levels of air pollutants in Jiuquan obtained from the fixed-site monitoring. The representativeness of the air pollution monitoring data would be very low, which could cause the results of this study to be inconsistent with foreign studies, especially for PM. However, up to now, researchers have traditionally relied on outdoor fixed-site monitoring data to reflect ambient exposures since they have had no other alternative. Consequently, raising the bar for exposure assessment in epidemiological research is both necessary and difficult. Second, we were unable to incorporate some factors including smoking, profession, outdoor time, and socioeconomic status since we did not have access to the necessary data.

## 5. Conclusions

Our results revealed that the effect of different air pollutants on COPD admissions varies drastically. In summary, this timeseries study demonstrates a positive association between short-term exposures to SO_2_, NO_2_, and CO and the increase in COPD admissions. However, PM_2.5_, PM_10_, and O_3_ exposure were not significantly associated with hospitalizations for COPD. Air pollutants have distinct impacts on gender, age, and season, as revealed by stratified analysis. Specifically, males, individuals aged ≥ 65 years, and the cold season had stronger adverse effects from an increase in NO_2_ and CO concentrations. To reduce the adverse impacts, strategies for controlling air pollution will include educating the public, using technological means to reduce sulfur emissions from various sources including the oil and petrochemical sectors, cutting back on diesel and fossil fuel consumption, and closely monitoring air pollution levels.

## Figures and Tables

**Table 1:** Descriptive statistics of daily hospital admissions for COPD, meteorological factors, and air pollution levels in Jiuquan from 2018 to 2019.

	$\overset{-}{X}$ ± S	Minimum	Percentile			Maximum
			*P*_25_	*P*_50_	*P*_75_	
**daily average hospital admissions**						
Total	8 ± 5	0	3	7	10	31
**Gender-specific (n)**						
Male	3 ± 2	0	1	3	5	15
Female	5 ± 3	0	2	3	6	23
**Age-specific (n)**						
<65 years	3 ± 2	0	1	2	4	16
≥65 years	5 ± 4	0	2	4	7	20
**Meteorological factors**						
Mean temperature (°C)	8.18 ± 7.16	−21.70	−1.70	9.90	19.13	27.30
Relative humidity (%)	46.49 ± 16.94	13.00	34.00	45.00	59.00	99.00
**Air pollutants**						
PM_2.5_ (μg/m^3^)	25.74 ± 15.56	8.00	16.00	22.00	32.00	166.00
PM_10_ (μg/m^3^)	78.41 ± 59.51	11.00	47.00	65.00	92.00	706.00
SO_2_ (μg/m^3^)	10.91 ± 5.00	4.00	8.00	10.00	13.00	36.00
NO_2_ (μg/m^3^)	22.55 ± 8.70	5.00	16.00	21.50	28.00	64.00
O_3_8h (μg/m^3^)	100.11 ± 22.61	46.00	82.00	102.00	117.00	168.00
CO (mg/m^3^)	0.57 ± 0.22	0.20	0.40	0.60	0.70	1.80

**Table 2:** Coefficients of Spearman’s correlation between daily air pollutants and meteorological factors in Jiuquan, 2018–2019.

Variables	PM_2.5_	PM_10_	SO_2_	NO_2_	CO	O_3_8h	Temperature	Humidity
PM_2.5_	1							
PM_10_	0.85 *	1						
SO_2_	0.45 *	0.32 *	1					
NO_2_	0.43 *	0.30 *	0.61 *	1				
CO	0.59 *	0.34 *	0.56 *	0.69 *	1			
O_3_8h	−0.39 *	−0.17 *	−0.37 *	−0.31 *	−0.35 *	1		
Temperature	−0.50 *	−0.25 *	−0.56 *	−0.34 *	−0.51 *	0.80 *	1	
Humidity	0.26 *	0.04	0.05	0.15 *	0.29 *	−0.33 *	−0.24 *	1

* *p* < 0.05.

## Data Availability

The datasets generated during and/or analyzed during the current study are not publicly available due to confidentiality agreements but are available from the corresponding author on reasonable request.

## Supplementary Materials

The following supporting information can be downloaded at: https://www.mdpi.com/article/10.3390/toxics12050364/s1, Figure S1: Association with a 10 μg/m^3^ increase in air pollutants for COPD admissions using single- and two-pollutant models; Table S1: Association between COPD admissions and ambient air pollutants: sensitivity varying controls in the regression time spline.
