# Supplementary material for: Short-Term Effects of Ambient Air Pollution on Chronic Obstructive Pulmonary Disease Admissions in Jiuquan, China
Source: Toxics. 2024 May 15;12(5):364. doi: 10.3390/toxics12050364 (PMC11125667; doi:10.3390/toxics12050364)
Supplement: Supplementary file 1 [file toxics-12-00364-s001.zip › toxics-2912423-supplementary.pdf]

Supplemental Materials

Short-Term Effects of Ambient Air Pollution on Chronic Obstructive Pulmonary Disease Admissions in Jiuquan, China

Figure S1. Association with a 10 μg/m^3 increase in air pollutants for COPD admissions using single, two pollutant models.

Table S1. Association between COPD admissions and ambient air pollutants: sensitivity varying controls in the regression time spline.

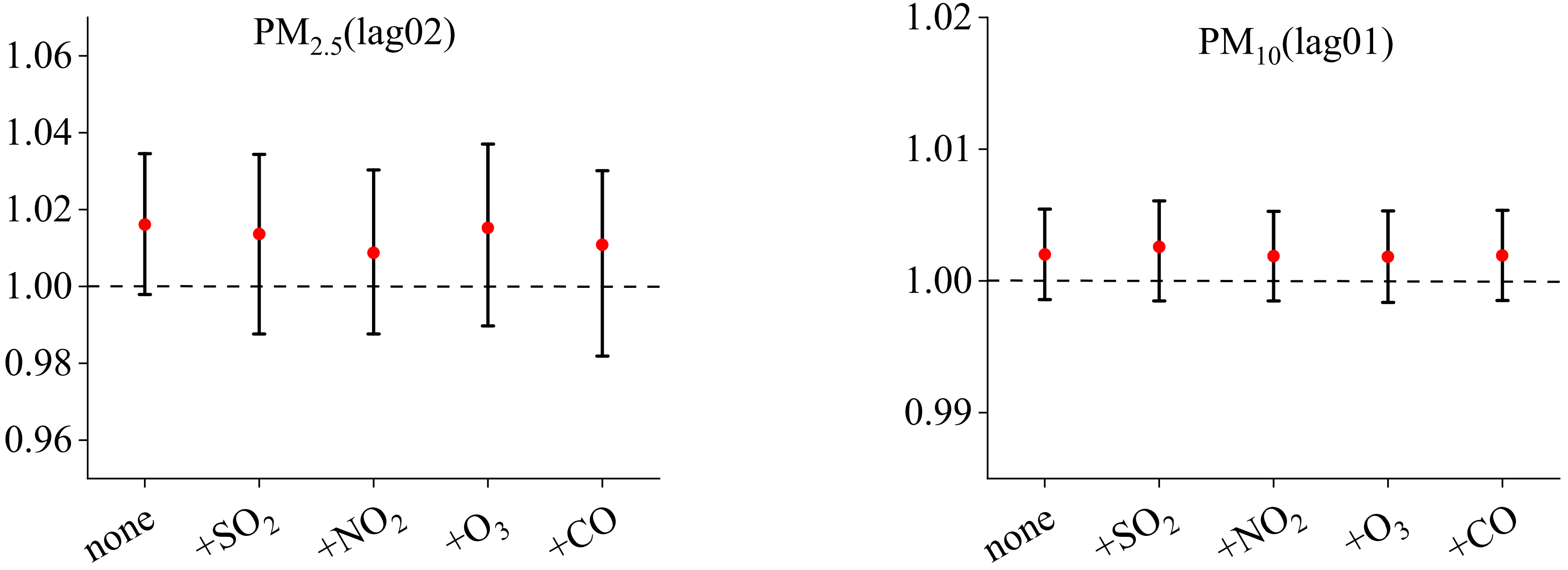

PM2.5(lag02)
1.06
1.04
1.02
1.00
0.98
0.96
none
+SO2
+NO2
+O3
+CO
PM10(lag01)
1.02
1.01
1.00
0.99
none
+SO2
+NO2
+O3
+CO

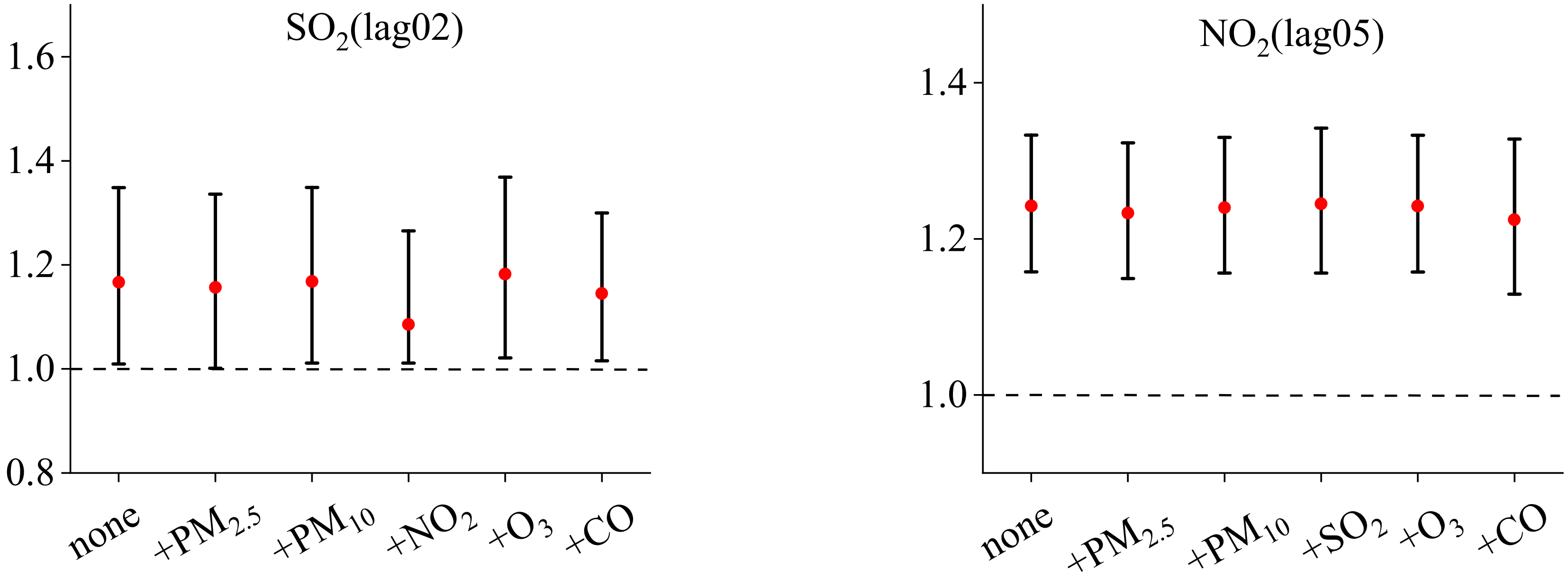

SO2(lag02)
1.6
1.4
1.2
1.0
0.8
none
+PM2.5
+PM10
+NO2
+O3
+CO
NO2(lag05)
1.4
1.2
1.0
none
+PM2.5
+PM10
+SO2
+O3
+CO

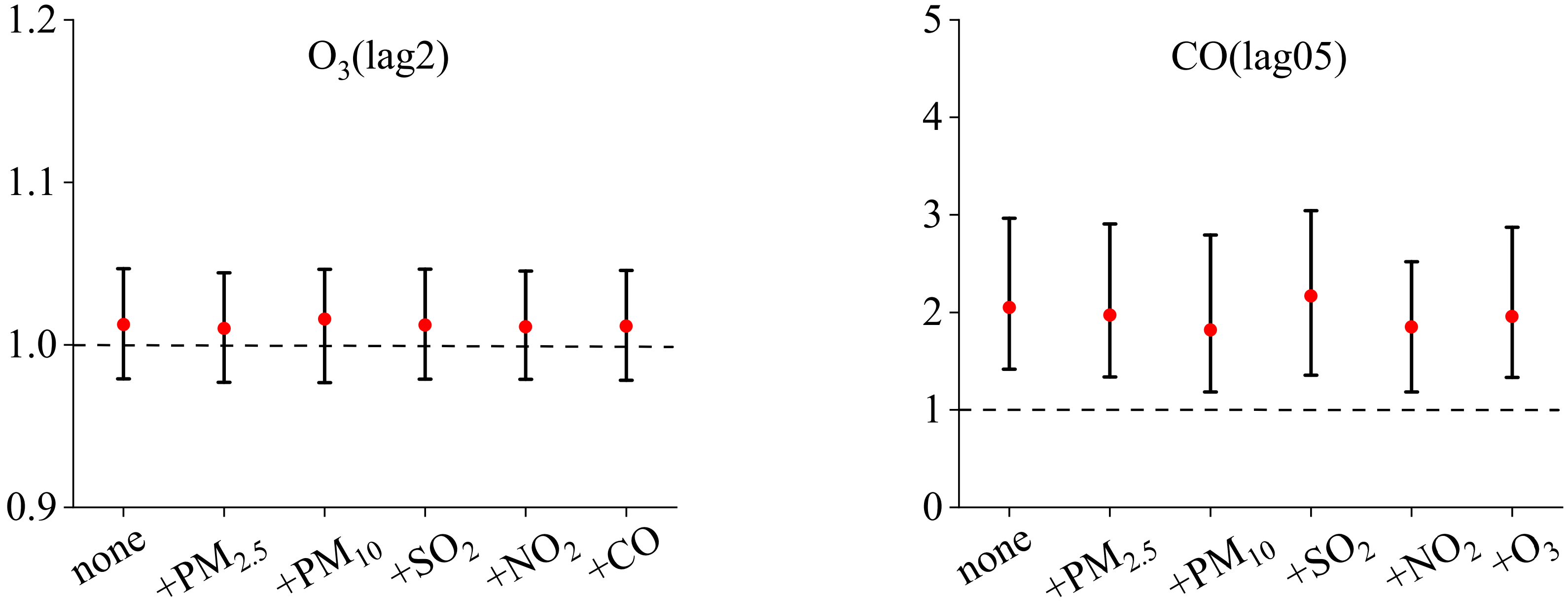

Figure S1. Association with a 10 μg/m³ increase in air pollutants for COPD admissions using single, two pollutant models.

Table S1. Association between COPD admissions and ambient air pollutants: sensitivity varying controls in the regression time spline.

	$PM_{2.5}$ (lag02)	SO_2 (lag02)	NO_2 (lag05)	O_3 (lag2)	CO (lag05)
df=6	1.017(0.999,1.036)	1.207(1.041,1.401)	1.282(1.191,1.380)	1.013(0.979,1.048)	2.132(1.479,3.074)
df=7	1.016(0.998,1.035)	1.167(1.009,1.348)	1.242(1.158,1.333)	1.012(0.979,1.047)	2.049(1.416,2.966)
df=8	1.017(0.999,1.035)	1.160(1.001,1.345)	1.262(1.173,1.357)	1.012(0.979,1.047)	2.114(1.453,3.075)
df=9	1.020(1.002,1.039)	1.143(0.984,1.327)	1.256(1.166,1.353)	1.015(0.981,1.05)	2.061(1.405,3.023)
df=10	1.017(0.999,1.036)	1.122(0.966,1.303)	1.253(1.163,1.349)	1.012(0.978,1.046)	1.952(1.333,2.86)

Note: The largest estimated effect of a given pollutant in the single-day lag model was used; Abbreviations: df, degree of freedom.
